# Prevalence of falls, incontinence, malnutrition, pain, pressure injury and restraints in home care: A narrative review

**DOI:** 10.1111/hsc.14021

**Published:** 2022-09-14

**Authors:** Lena Maria Lampersberger, Silvia Bauer, Selvedina Osmancevic

**Affiliations:** ^1^ Institute of Nursing Science Medical University of Graz Graz Austria

**Keywords:** falls, home care, incontinence, malnutrition, pain, pressure injury, restraints

## Abstract

Global demographic changes and the strategy of ‘ageing in place’ will increase the importance of home care in the future. To deliver safe and high‐quality care, clinical data on nursing‐sensitive indicators and transparency are needed. A comprehensive narrative review of the literature was conducted to describe the prevalence and incidence of nursing‐sensitive indicators, namely, falls, incontinence, malnutrition, pain, pressure injury and restraints in home care. A literature search was carried out in May 2021 in PubMed and CINAHL, and 28 studies were included. Data were extracted using two extraction tables designed for this review. Prevalence and incidence rates varied widely and internationally within each indicator. The prevalence range for falls was 4.8%–48%; urinary incontinence, 33.7%–62.5%; malnutrition, 20%–57.6%; pain, 6.5%–68.5%; pressure injury, 16%–17.4% and physical restraints, 5%–24.7%. Due to various measurements and different instruments, the rates are not comparable. The use of standardised measurement and risk assessment tools to assess nursing‐sensitive indicators in home care is needed to implement suitable interventions and to prevent these indicators.

**What is known about this topic**

- Patient safety enables the provision of high‐quality care in all healthcare settings.
- Measuring nursing‐sensitive indicators like malnutrition or falls increases patient safety and thus quality of care.
- Although nursing‐sensitive indicators are possibly preventable, the prevalence is still high.

**What this paper adds**

- Falls and malnutrition were measured most often in home care.
- The prevalence of falls in home care ranged from 4.8% to 48% and malnutrition from 4.2% to 57.6%.
- A standardised measurement of nursing‐sensitive indicators in home care is recommended to increase the quality of care by enabling the initiation of preventive measures and interventions where needed.

## 1 INTRODUCTION

Older adults who need care and assistance prefer to live independently and stay in their own homes (TNS Opinion & Social, 2007). This is also referred to as ‘ageing in place’ (OECD, 2005; Pani‐Harreman et al., 2021). Home care (HC) can be defined as care provided by professional carers within the clients' own homes (Genet et al., 2012). It can be provided on a short‐term or long‐term care basis and the services provided may be preventive, acute, rehabilitative or palliative (Genet et al., 2012; OECD, 2005). Informal care which is provided by spouses, family members, friends and volunteers additionally exists, and this accounts for a large proportion of complementary care (Garms‐Homolová, 2008; Genet et al., 2012). In order to support the older adults' preference to ‘age in place’, informal caregivers constitute an important pillar in HC (Genet et al., 2012). Due to demographic changes and restrictions in the availability of informal caregivers (e.g. due to women's occupations) (Mestheneos & Triantafillou, 2005), the use of HC services becomes even more important and is expected to increase in the near future. Older adults are not the sole recipients of HC, also children and adults are in need of the services, for example, after hospitalisation or when suffering from disabilities (Genet et al., 2012) In 2020, for instance, 8% of HC clients in Austria were under the age of 60 (Statistik Austria, 2021). Hence, it is of the utmost importance to ensure that HC services provide safe and high‐quality care for people so that they can be effectively looked after at home. According to the World Health Organisation (WHO), patient safety is defined as ‘the absence of preventable harm to a patient during the process of health care and reduction of risk of unnecessary harm associated with health care to an acceptable minimum’ (WHO, 2020). Patient safety is essential to ensure the quality of care, not only in healthcare institutions (Ammouri et al., 2015) but also in the HC setting.

One possible way to provide qualitative care is to collect and interpret data relevant to nursing care, as analyses of these data increase the transparency of care. These measurements and the increased transparency draw attention towards the areas of nursing care that are of good quality but also towards areas that need further improvements (Donabedian, 1988). To achieve this, the right measurements of indicators reflecting nursing care need to be taken. Several attempts have been made to give recommendations about appropriate indicators that can be used to measure and operationalise nursing care (Doran et al., 2011; Maben et al., 2012). These so‐called ‘nursing‐sensitive indicators’, therefore, have become increasingly important in nursing care quality and performance measurement, not only in the acute care setting but also in the HC setting (Heslop et al., 2014). Nursing‐sensitive indicators which should be measured and are broadly and internationally recognised in specific areas of nursing care include (facility‐acquired) pressure injury prevalence, pressure injury risk, pain, nutrition, infection prevention, restraints and patient falls (Dubois et al., 2013; Joseph & Samson, 2016; Maben et al., 2012). Dubois et al. (2013) developed a pool of indicators sensitive to various aspects of nursing care—the Nursing Care Performance Framework. Of the three main categories, the third category (producing changes in patients' conditions) includes indicators such as the prevalence of falls, incontinence, malnutrition, pain, pressure injury and restraints. These should be measured to operationalise nursing care (Dubois et al., 2013). The National Prevalence Measurement of Care Quality (LPZ study) is based on the framework of Dubois et al. (2013) and measures nursing‐sensitive indicators (falls, incontinence, malnutrition, pain, pressure injury and restraints), as they provide an insight into nursing quality (Halfens et al., 2013; van Nie‐Visser et al., 2013). These nursing‐sensitive indicators are potentially preventable (Soban et al., 2011) but still highly prevalent in different settings (Wadensten et al., 2011). Several international studies have measured nursing‐sensitive indicators in the HC setting. A recent study by Neziraj et al. (2021) found that the prevalence of pressure injuries was 27.9%, the prevalence of malnutrition was 56.3% and the prevalence of falls was 74.5% in clients receiving HC in southern Sweden (Neziraj et al., 2021). Furthermore, the prevalence of urinary incontinence in HC clients was described to be 33.7% in Canada (Northwood et al., 2021). Most of the previously performed studies (e.g. Brett et al., 2020; Geurden et al., 2015; Northwood et al., 2021) describe only some of the nursing‐sensitive indicators or do not focus primarily on the HC setting. A narrative review of several common nursing‐sensitive indicators will provide a detailed overview of the status quo of the quality of nursing in HC in different care systems. Policy makers, academics and those responsible for service delivery can access updated prevalence or incidence rates on common nursing‐sensitive indicators. Furthermore, this review can show the frequency in which each indicator is measured in different regions, which indicates research gaps in HC that can, in turn, prompt HC organisations to further use prevalence rates to improve nursing practice in the long run. Therefore, the aim of conducting this review was to describe the current state of the art regarding the prevalence and incidence of common nursing‐sensitive indicators in HC in the international literature.

## 2 MATERIALS AND METHODS

A comprehensive narrative review of the literature was undertaken to identify and synthesise included studies. A summary of the current literature was not performed in an explicitly systematic way, but a systematic literature review was conducted, a specific research question was asked, and a narrative summary of included studies is provided (Baethge et al., 2019).

A literature search in the online databases PubMed and Cumulative Index to Nursing and Allied Health Literature (CINAHL) was carried out in May 2021 using a range of search terms to identify relevant studies. In addition, we hand searched the references of the identified articles to detect additional studies and conducted a search in Google Scholar, where the first 10 pages were searched. For each care problem, a search string was developed for PubMed and translated to CINAHL (see Table 1). We designed the search strategy using keywords, their synonyms and, if these were available, MeSH Terms (Medical Subject Headings) in PubMed or MH (Major Headings) in CINAHL. For Google Scholar, the search string was adapted accordingly.

**TABLE 1:** Search terms for each variable

	PubMed	CINAHL
Prevalence/incidence	Prevalen* (+MeSH) OR inciden* (+MeSH) OR epidemiolog* (+MeSH Subh) OR occurrence* OR rate*	Prevalen* (+MH) OR inciden* (+MH) OR epidemiolog* (+MH) OR occurrence* OR rate*
*AND*		
Home care	‘home care’ OR ‘home healthcare’ OR ‘home nursing’ (+MeSH) OR home care service (+MeSH)	‘home care’ OR ‘home healthcare (+MH)’ OR ‘home nursing’ (+MH) OR ‘home care service*’
*AND*		
Pain	Pain (+MeSH) OR ache	Pain (+MH) OR ache
Falls	fall*OR accidental falls (+MeSH)	fall* OR ‘accidental fall*’ (MH)
Restraints	restraint* OR seclusion* OR restraints, physical (+MeSH) OR ‘pharmacological restraint*’ OR ‘chemical restraint’ OR ‘patient seclusion*’	‘restraint, chemical +MH’ OR ‘restraint, physical’ + MH OR restraint* OR seclusion* OR ‘patient seclusion’ + MH OR ‘pharmacological restraint*’
Incontinence	incontinen* OR urinary incontinence (MeSH) OR faecal incontinence (MeSH)	incontinen* OR incontinence MH OR urinary incontinen* (+MH) OR faecal incontinen* (+MH)
Malnutrition	Malnutrition (+MeSH) OR ‘poor diet’ OR ‘poor nutrition’ OR ‘undernutrition’ OR ‘nutrition’ OR ‘nutritional status’ (+MeSH)	malnutrition (+MH) OR ‘poor diet’ OR ‘poor nutrition’ OR ‘undernutrition’ OR ‘nutrition’ (+MH) OR ‘nutritional status’
Pressure injury	pressure ulcer (+MeSH) OR ‘pressure injury’ OR ‘pressure sore’ OR ‘decubitus ulcer’	pressure ulcer (+MH) OR ‘pressure injury’ OR pressure sore OR decubitus ulcer

### 2.1 Inclusion and exclusion criteria

The Condition, Context, Population (CoCoPop) framework, which was developed for systematic reviews of epidemiological studies reporting prevalence and incidence rates (Munn et al., 2015), was used to create the research questions. This framework is recommended by the Joanna Briggs Institute (JBI) for systematic reviews of prevalence and incidence (Munn et al., 2020), as the population, intervention, control and outcome (PICO) framework is not appropriate for research questions regarding prevalence and incidence rates. In these types of questions, no interventions or outcomes are measured, nor can an effect be measured by a control intervention (Munn et al., 2015, 2020). This framework was also chosen to create specific research questions and to develop the search approach for this narrative review. Building the CoCoPop frame, the six nursing‐sensitive indicators represented the conditions, the HC setting represented the context, and HC clients 18 years and older represented the population. Therefore, the following research questions were developed using the CoCoPop:

1. What is the prevalence/incidence of falls in adult HC clients?
2. What is the prevalence/incidence of restraints in adult HC clients?
3. What is the prevalence/incidence of incontinence in adult HC clients?
4. What is the prevalence/incidence of malnutrition in adult HC clients?
5. What is the prevalence/incidence of pain in adult HC clients?
6. What is the prevalence/incidence of pressure injuries in adult HC clients?

We included studies that examined the prevalence and incidence of falls, restraints, incontinence, malnutrition, pain or pressure injuries. All types of measurements of the prevalence and incidence of the six nursing‐sensitive indicators were included. Studies with the setting HC were included in the review. Studies that took place exclusively in nursing homes, hospitals or other healthcare institutions were excluded. Participants included in the studies had to be 18 years or older in order to include all adult HC clients. Home palliative care was not included in this review. Studies included had to be published in the last 10 years (January 2011–May 2021) to include current studies. In addition, the search was limited to reports in English or German language due to the limited linguistic proficiency of the authors. Only published studies were included in this review. Conference abstracts, study protocols, policy papers and letters were not included.

### 2.2 Study selection

Study selection was conducted in four rounds by one author (L.M.L.). First, the duplicates were removed, and then the titles and abstracts were screened. Next, the full texts were screened for inclusion based on the eligibility criteria. In case of uncertainty, the study was discussed with one other author (S.O.) until a consensus was found.

To evaluate the quality of the selected studies, each study was critically evaluated according to the key results, methods used to assess the prevalence as well as the quality and interpretation of the results (Ferrari, 2015).

### 2.3 Data collection and synthesis

The data collection and synthesis steps were performed by one author (L.M.L.). Two data extraction tables were designed and used to extract relevant data. In the first table, data were collected on the characteristics of the selected study, including the author, year, design, country the study was conducted, information about what nursing‐sensitive indicator was assessed, sample size and sociodemographic characteristics of the participants (Table 2). In the second table, information about the measurement of nursing‐sensitive indicators as well as prevalence and incidence rates were collected. Regarding the measurement of nursing‐sensitive indicators, either the used instrument or routine data (e.g. data that are routinely collected by nursing staff like weight or height of clients) is presented (Table 3).

**TABLE 2:** Study characteristics and demographic data

Reference (year) and country	Design	Nursing‐sensitive indicators	Number of participants in HC	Mean age	Female (%)
Adıgüzel and Acar‐Tek (2019), Turkey	Cross‐sectional descriptive study	Prevalence of malnutrition	209	76.2 (±17.39)	56.9
Brett et al. (2020), Australia	Longitudinal, observational study	Prevalence of falls	1596	82 (±7.8)	66
Burton et al. (2018), Australia	Cross‐sectional descriptive study	Prevalence of falls	1991	81.1 (±8.2)	74.6
Cereda et al. (2016), Europe, Asia, USA, Australia and Africa	Systematic review and meta‐analysis	Prevalence of malnutrition	N/A	N/A	N/A
da Silva Gabriel Capeletto et al. (2021), Brazil	Cross‐sectional, observational study	Prevalence of restraints	162	Largest age group: 69–76 years	60.5
Doran et al. (2013), Canada	Retrospective cohort design in four different provinces	Incidence of falls, incontinence and pressure injury	451,167	68.3–74.6	58.8–63.2
Geurden et al. (2015), Belgium	Cross‐sectional study	Prevalence of malnutrition	100	75.2 (±17)	78
Johansen et al. (2015), Norway	Cross‐sectional study	Prevalence of pressure injury	31	67% > 80 years	N/A
Kiesswetter et al. (2020), Germany	Secondary data analysis	Prevalence of malnutrition	335	80.9 (±7.7)	63.6
Kiesswetter et al. (2014), Germany	Multicenter, prospective observational study	Prevalence of malnutrition	309	80.9 (±7.9)	63.8
Lahmann et al. (2016), Germany	Multicenter cross‐sectional study	Prevalence of malnutrition	878	78.5 (±12.2)	62.9
Leiske et al. (2015), Germany	Multicenter cross‐sectional study	Prevalence of pain	878	78.8 (±12.1)	62.9
Meijers et al. (2012), the Netherlands	Secondary data analysis	Prevalence of malnutrition	2971	81.5 (±7)	67.9
Mengelers et al. (2020), the Netherlands and Belgium	Secondary data analysis	Prevalence of restraints	868^a^	82 (±6.7)	60.1
Meyer et al. (2020), Australia	Retrospective data analysis	Prevalence of falls	1574^b^	Fallers: 81.6 (±7.8)	Fallers: 45
Neziraj et al. (2021), Sweden	Retrospective cross‐sectional study	Prevalence of falls, malnutrition and risk of pressure injury	1692	84.5 (±7.8)	66
Northwood et al. (2021), Canada	Cross‐sectional study	Prevalence of incontinence	118,519^b^	80.7 (±7.8)	57.6
Rist et al. (2012), Australia	Cross‐sectional study	Prevalence of malnutrition	235	82 (±7)	52.8
Rocha do Carmo et al. (2020), Brazil	Cross‐sectional Study	Prevalence of falls	131	66.8 (±21.2)	55
Scheepmans et al. (2017), Belgium	Cross‐sectional study	Prevalence of restraints	6397	80.6 (±7.8)	66.8
Scheepmans et al. (2018), Europe, Japan, USA	Systematic review	Prevalence of restraints	N/A	N/A	N/A
Schildmeijer et al. (2018), Sweden	Retrospective record review	Prevalence of falls, pain and pressure injury	600	80.5 median (range 20–99)	53.3
Schilp et al. (2012), the Netherlands	Cross‐sectional study	Prevalence of malnutrition	814	81.6 (±7.4)	69.3
Suhr and Lahmann (2018), Germany	Cross‐sectional study	Prevalence of incontinence	923	80.4 (±11.2)	65.5
van der Pols‐Vijlbrief et al. (2016), the Netherlands	Cross‐sectional study	Prevalence of malnutrition	300	81.7 (±7.6)	68.3
Vikman et al. (2011), Sweden	Prospective cohort study	Prevalence and incidence of falls	614	81.8 (±6.8)	67
Xu et al. (2018), China	Cross‐sectional study	Prevalence of pain	323	77.3 (±7.1)	72.7

Abbreviations: HC, Home care; N/A, not applicable.

^a^ Only HC clients with dementia.

^b^ Only HC clients with diabetes mellitus.

**TABLE 3:** Prevalence or incidence rates of the nursing‐sensitive indicators in the included studies

Study	Measurement of nursing‐sensitive indicators	Prevalence
*Falls*		
Brett et al. (2020)	Routine data	One or more falls during the last 19 months: 4.8% Unique fall: 84%
Burton et al. (2018)	Self‐developed questionnaire	Falls in the year before the survey: 48% Falls in the last month: 32.7% Unique fall: 41.9%
Doran et al. (2013)	RAI‐HC Routine data	Injurious fall incidence (unadjusted): 1.7%–3.6%
Meijers et al. (2012)	Meijers definition of malnutrition (Meijers et al., 2009)	Falls during the last 30 days: 12.2%
Meyer et al. (2020)	Routine data	Falls during the last 6 months: 30.6%
Neziraj et al. (2021)	MNS MNA‐SF DFRI	Risk of falls: 63.7%
Rocha do Carmo et al. (2020)	Self‐developed questionnaire	Falls in the year before the survey: 43.5% Unique fall: 49.1% Multiple falls: 50.9%
Schildmeijer et al. (2018)	Routine data	Falls in a 90‐day period: 18.5%
Vikman et al. (2011)	Routine data Self‐developed questionnaire	Prevalence: 20% fell during 1 year Unique fall: 61% Multiple falls: 39% Incidence: 626 per 1000 per year
*Incontinence*		
Doran et al. (2013)	RAI‐HC Routine data	Urinary incontinence: 26.3–27.6% Faecal incontinence: 6.8–10.3%
Northwood et al. (2021)	RAI‐HC	Urinary incontinence: 33.7%
Suhr and Lahmann (2018)	ICIQ‐SF	Urinary incontinence: 65.5%
*Malnutrition*		
Adıgüzel and Acar‐Tek (2019)	Self‐developed questionnaire MNA	Malnutrition: 52.6% Risk of malnutrition: 30.1% Underweight (BMI): 7.7%
Cereda et al. (2016)	MNA	Malnutrition: 8.7% Risk of malnutrition: 47.5%
Geurden et al. (2015)	MUST	Risk of malnutrition: 29%
Kiesswetter et al. (2014)	MNA‐LF MNA‐SF	Malnutrition MNA‐SF: 14.9% MNA‐LF: 13.6% Risk of malnutrition MNA‐SF: 41.1% MNA‐LF: 57.6%
Kiesswetter et al. (2020)	Self‐developed questionnaire Routine data	Malnutrition (weight loss): 15.8%
Lahmann et al. (2016)	Barthel‐Index Item feeding MNA‐SF MUST Routine data Other (clinical judgement)	Malnutrition MNA‐SF: 4.8% Risk of malnutrition MUST: 4.2% middle risk MUST: 6.8% high risk MNA‐SF: 20% Underweight (BMI): 8.7% Cachectic: 10.2%
Meijers et al. (2012)	Meijers definition of malnutrition (Meijers et al., 2009)	Malnutrition: 16.2%
Neziraj et al. (2021)	MNS MNA‐SF DFRI	Risk of malnutrition MNA‐SF: 47.3%
Rist et al. (2012)	Instrument MNA Other (anthropometric measurements)	Malnutrition: 8.1% Risk of malnutrition: 34.5% Underweight (BMI): 19.1%
Schilp et al. (2012)	SNAQ^65+^	Malnutrition: 34.8% Risk of malnutrition: 9.2%
van der Pols‐Vijlbrief et al. (2016)	SNAQ^65+^	Malnutrition: 31.7% Risk of malnutrition: 8%
*Pain*		
Leiske et al. (2015)	VAS	Pain: 68.5% Mean pain intensity: 2.9 ± 2.8
Schildmeijer et al. (2018)	Routine data	Pain: 6.5%
Xu et al. (2018)	RAI‐HC RAI‐LTCF	Moderate pain: 17.65% Daily severe pain: 19.81%
*Pressure injury*		
Doran et al. (2013)	RAI‐HC Routine data	Pressure injury incidence (unadjusted): 0.6–1.2% Pressure Injury ≥ stage 2 incidence (unadjusted): 0.06–0.1%
Johansen et al. (2015)	Braden Scale Self‐developed questionnaire	Pressure Injury: 16% Risk of Pressure injury Mild risk: 21% Moderate risk: 10%
Neziraj et al. (2021)	MNS MNA‐SF DFRI	Risk of pressure injury: 13.8%
Schildmeijer et al. (2018)	Routine data	Pressure injury: 17.4% Stage 1: 38.7% Stage ≥2: 61.3%
*Restraints*		
da Silva Gabriel Capeletto et al. (2021)	Self‐developed questionnaire Other (observations)	Physical restraints: 13% Environmental restraints (access to other rooms/outdoors): 85.7%
Mengelers et al. (2020)	Self‐developed questionnaire	Physical restraints: 18.5% Psychotropic medication: 40.7% Non‐consensual care (e.g. locking a door, removing walking aids): 82.7%
Scheepmans et al. (2017)	Self‐developed questionnaire Routine data	(Physical) restraints in the month before the survey (e.g. bed against wall, adaptation of the house): 24.7%
Scheepmans et al. (2018)	N/A	(Physical) restraints: 5–24.7%

Abbreviations: BMI, Body Mass Index; DAD, Discharge Abstract Database; DFRI, Downtown Fall Risk Index; HC, home care; ICIQ, Urinary Incontinence Form; LF, long form; LTCF, long‐term care facilities; MNA, Mini Nutritional Assessment; MNS, Modified Norton Scale; MUST, Malnutrition Universal Screening Tool; N/A, not applicable; RAI‐HC, Resident Assessment Instrument for Home Care; SF, short form; SNAQ^65+^, Short Nutritional Assessment Questionnaire; VAS, Visual Analogue Scale.

## 3 RESULTS

**FIGURE 1:**
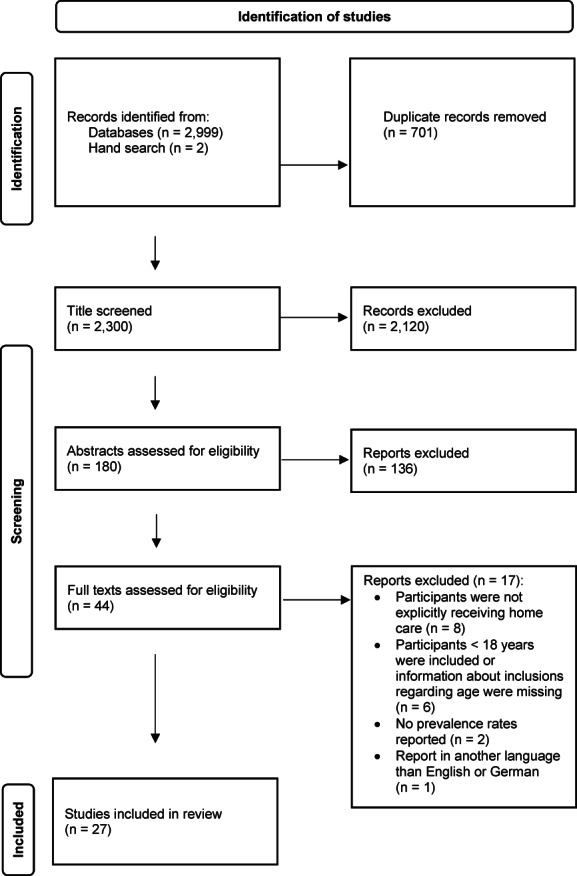
PRISMA flow diagram of the study selection process, adapted from Page et al. (2021).

An overview of the process of study selection and reasons for exclusion is shown in Figure 1. Our search identified 3001 studies. Before title screening was performed, 701 duplicates were removed; consequently, 2300 titles were screened and 2120 were excluded. Out of the 180 abstracts screened, 136 were found not to be eligible for this literature review. After applying the eligibility criteria to the full‐text versions of 44 studies, 17 studies were excluded for various reasons, for example participants were not explicitly receiving HC or were younger than 18 years. A final selection of 27 studies was included in this literature review (Figure 1).

An overview of the characteristics of the individual studies is presented in Table 2. From 27 included studies, the majority (*n* = 16; 59.3%) was conducted in Europe (Adıgüzel & Acar‐Tek, 2019; Geurden et al., 2015; Johansen et al., 2015; Kiesswetter et al., 2020, 2014; Lahmann et al., 2016; Leiske et al., 2015; Meijers et al., 2012; Mengelers et al., 2020; Neziraj et al., 2021; Scheepmans et al., 2017; Schildmeijer et al., 2018; Schilp et al., 2012; Suhr & Lahmann, 2018; van der Pols‐Vijlbrief et al., 2016; Vikman et al., 2011). The others were conducted in Australia (Brett et al., 2020; Burton et al., 2018; Meyer et al., 2020; Rist et al., 2012), Canada (Doran et al., 2013; Northwood et al., 2021), Brazil (da Silva Gabriel Capeletto et al., 2021; Rocha do Carmo et al., 2020) and China (Xu et al., 2018). Two studies were systematic reviews which included samples from Europe, Asia, America, Australia and Africa (Cereda et al., 2016) and Europe, Asia and America (Scheepmans et al., 2018).

In total, the six nursing‐sensitive indicators of falls, incontinence, malnutrition, pain, pressure injury and restraints were reported 34 times in the 27 included studies. The most frequently measured indicator in HC was malnutrition (*n* = 11; Adıgüzel & Acar‐Tek, 2019; Cereda et al., 2016; Geurden et al., 2015; Kiesswetter et al., 2014, 2020; Lahmann et al., 2016; Meijers et al., 2012; Neziraj et al., 2021; Rist et al., 2012; Schilp et al., 2012; van der Pols‐Vijlbrief et al., 2016), followed by falls (*n* = 9) (Brett et al., 2020; Burton et al., 2018; Doran et al., 2013; Meijers et al., 2012; Meyer et al., 2020; Neziraj et al., 2021; Rocha do Carmo et al., 2020; Schildmeijer et al., 2018; Vikman et al., 2011). Prevalence and incidences regarding restraints (da Silva Gabriel Capeletto et al., 2021; Mengelers et al., 2020; Scheepmans et al., 2017, 2018) and pressure injury (Doran et al., 2013; Johansen et al., 2015; Neziraj et al., 2021; Schildmeijer et al., 2018) were reported in four studies. The prevalence of pain (Leiske et al., 2015; Schildmeijer et al., 2018; Xu et al., 2018) and incontinence was reported in three studies (Doran et al., 2013; Northwood et al., 2021; Suhr & Lahmann, 2018) (Table 3).

The majority of the included studies were cross‐sectional (*n* = 16; 59.3%). The sample sizes in the included studies varied between 31 (Johansen et al., 2015) and 118,519 (Northwood et al., 2021) participants. The mean age of the clients who participated in the included studies ranged from 66.8 years (Rocha do Carmo et al., 2020) to 84.5 years (Neziraj et al., 2021), and the majority of these were female (Table 2).

### 3.1 Falls

In studies reporting fall prevalence and incidence, the falls in the last 30 days (Meijers et al., 2012) to 19 months (Brett et al., 2020) were assessed. Routine data and self‐developed questionnaires were used most frequently to measure falls. The prevalence rates of one or more falls in HC clients ranged from 4.8% (Brett et al., 2020) to 48% (Burton et al., 2018). The incidence of falls was 626 per 1000 per year (Vikman et al., 2011). The prevalence of risk of falls was reported in one study by Neziraj et al. (2021) and was 63.7% in HC clients (Table 3).

### 3.2 Incontinence

To assess the prevalence and incidence of incontinence, the Resident Assessment Instrument for Home Care (RAI‐HC) was mainly used. In addition, the Urinary Incontinence Form (ICIQ) was used by Suhr and Lahmann (2018). Urinary incontinence was reported far more frequently compared to faecal incontinence. The prevalence of urinary incontinence was found to be between 33.7% (Northwood et al., 2021) and 62.5% (Suhr & Lahmann, 2018), with incidences ranging between 26.3% and 27.6% (Doran et al., 2013). The incidence of faecal incontinence was reported in one study by Doran et al. (2011) and included the incidence rates for two consecutive years and in four different regions in Canada, ranging from 6.8% to 10.3% (Table 3).

### 3.3 Malnutrition

Different measuring tools were used to assess malnutrition or the risk of malnutrition: the Mini Nutritional Assessment (MNA) short and/or long form (Adıgüzel & Acar‐Tek, 2019; Cereda et al., 2016; Kiesswetter et al., 2014; Lahmann et al., 2016; Neziraj et al., 2021; Rist et al., 2012), the Body Mass Index (BMI) (Adıgüzel & Acar‐Tek, 2019; Lahmann et al., 2016; Rist et al., 2012), the Short Nutritional Assessment Questionnaire (SNAQ^65+^) (Schilp et al., 2012; van der Pols‐Vijlbrief et al., 2016), the Malnutrition Universal Screening Tool (MUST) (Geurden et al., 2015; Lahmann et al., 2016), and clinical judgement if the clients were cachectic (Lahmann et al., 2016). The prevalence of malnutrition assessed with an instrument (MUST, MNA, SNAQ^65+^, definition by Meijers et al. (2009)) ranged between 4.8% (Lahmann et al., 2016) and 52.6% (Adıgüzel & Acar‐Tek, 2019), and the risk of malnutrition spanned from 4.2% (Lahmann et al., 2016) to 57.6% (Kiesswetter et al., 2014). Between 7.7% (Adıgüzel & Acar‐Tek, 2019) and 19.1% (Rist et al., 2012) of the HC clients were underweight with regard to BMI, and 10.2% were cachectic according to clinical judgement (Lahmann et al., 2016). (Table 3).

### 3.4 Pain

Instruments used to assess pain in HC clients were the Visual Analogue Scale (VAS) (Leiske et al., 2015), the RAI‐HC (Xu et al., 2018) and routine data (Schildmeijer et al., 2018). The prevalence of pain spanned from 6.5% (Schildmeijer et al., 2018) to 68.5% (Leiske et al., 2015). According to Xu et al. (2018), 17.65% of the participants experienced moderate pain and 19.81% experienced severe pain daily. With regard to the VAS, the mean pain intensity was 2.9 ± 2.8 on a scale from 0 to 10; 0 indicates the absence of pain and 10 indicates the most severe pain (Leiske et al., 2015) (Table 3).

### 3.5 Pressure injury

To assess the prevalence and incidence of pressure injuries, different instruments (RAI‐HC, Braden Scale) as well as routine data and self‐developed questionnaires were used. In the studies investigating pressure injuries in HC clients, the prevalence of pressure injuries was 16% (Johansen et al., 2015) and 17.4% (Schildmeijer et al., 2018). The incidence of pressure injury ranged from 0.6% to 1.2% (Doran et al., 2013) (Table 3). Table 3 also shows distinctions between pressure injury stages. The European Pressure Ulcer Advisory Panel, National Pressure Injury Advisory Panel, & Pan Pacific Pressure Injury Alliance (2019) defined four stages of pressure injuries, from stage I as non‐blanchable erythema and up to stage IV as full‐thickness tissue loss.

### 3.6 Restraints

Self‐developed questionnaires were mainly used to collect data on the prevalence and incidence of restraints. Overall, the prevalence of any type of restraints ranged between 5% (Scheepmans et al., 2018) and 85.7% (da Silva Gabriel Capeletto et al., 2021). The prevalence of physical restraints ranged from 5% (Scheepmans et al., 2018) to 24.7% (Scheepmans et al., 2018; Scheepmans et al., 2017). The prevalence of psychotropic medication ranged up to 40.7% (Mengelers et al., 2020). Environmental restraints, such as blocking access to another room or to the outdoors according to da Silva Gabriel Capeletto et al. (2021), were used in 85.7% of HC clients (da Silva Gabriel Capeletto et al., 2021), and non‐consensual care, such as removing a walking aid or locking the door according to Mengelers et al. (2020), was used in 82.7% of cases (Mengelers et al., 2020; Table 3).

## 4 DISCUSSION

This review was carried out to describe the current state of the art regarding the prevalence of six common nursing‐sensitive indicators (falls, incontinence, malnutrition, pain, pressure injury and restraints) in HC. Malnutrition and falls were the most frequently investigated indicators in HC, whereas incontinence and pain were the topics subject to the least research. The prevalence rates for falls ranged between 4.8% (Brett et al., 2020) and 48% (Burton et al., 2018). For malnutrition, the prevalence measured with different instruments was between 4.8% (Lahmann et al., 2016) and 52.6% (Adıgüzel & Acar‐Tek, 2019), and the risk of malnutrition ranged between 4.2% (Lahmann et al., 2016) and 57.6% (Kiesswetter et al., 2014).

The prevalence rates presented in the literature for the six nursing‐sensitive indicators vary widely. The participants assessed in the included studies were not homogeneous, and some studies examined HC clients with a specific diagnosis due to their higher risk for a specific nursing‐sensitive indicator. Mengelers et al. (2020) examined restraints in HC clients with dementia; Meyer et al. (2020), falls in HC clients with diabetes mellitus; and Northwood et al. (2021), incontinence in HC clients with diabetes mellitus, which may have influenced the wide range of prevalence rates. People with dementia, for example, have a higher risk of falls (Allan et al., 2009) as do people with diabetes mellitus (Yang et al., 2016). Since comorbidities can influence on an individual's risk for specific care problems, many HC clients are also affected by more than one care problem which can lead to higher utilisation of HC (Cegri et al., 2020). For instance, in the study of Neziraj et al. (2021), 30% of the participants had two health risks, a risk of pressure injuries and a risk of malnutrition, or a risk of falls.

Some studies included participants 18 years and older (e.g. Geurden et al., 2015; Johansen et al., 2015; Schildmeijer et al., 2018), and others included more specific older adults, most of whom were 65 years and older (e.g. Brett et al., 2020; Neziraj et al., 2021; Schilp et al., 2012), which also results in a variation of the mean age of participants in the included studies. This variation in the mean age may account for some differences in findings, as old age is a risk factor for the prevalence of falls (Brett et al., 2020), incontinence (Suhr & Lahmann, 2018), malnutrition (Schilp et al., 2012), pain (Leiske et al., 2015), pressure injury (Cai et al., 2019) and frailty (Feng et al., 2017). The variations in prevalence may also be due to the differences in sample characteristics, such as care dependency and multi‐morbidity. Care dependency and multi‐morbidity considerably influence, for example the prevalence of malnutrition in different countries. Among others, dementia greatly influences the occurrence of malnutrition, meaning that people with dementia have a specifically high risk of becoming malnourished (Mole et al., 2018).

In the included studies, differences in measurements were also prevalent. With regard to the nursing‐sensitive indicator of malnutrition, widely known and comprehensively tested tools were used, but only the MUST is recommended for use in HC (Kondrup et al., 2003). However, the MUST was only used in two identified studies. The different measurement instruments and the use of instruments that were not primarily intended for use in the HC setting may account for the substantial differences in malnutrition prevalence rates. Nevertheless, the high rates as well as the high number of identified studies investigating this topic underline the importance of malnutrition in home care.

The study by Vikman et al. (2011) showed that fall incidence is strongly correlated with the number of home help services received and with dependency on activities of daily living. The risk of falls increases by 7% per each increased weekly hour of received home care. As shown by van der Pols‐Vijlbrief et al. (2016), malnutrition is also shown to be associated with dependency on activities of daily living. Purchasing food or preparing meals can be difficult for HC clients (van der Pols‐Vijlbrief et al., 2016); thus, nurses need to be aware of their clients' possible difficulties in meeting their needs. In order to be aware of the client's needs, their resources and support needs must be assessed. For this purpose, the measurement tools need to be selected carefully in order to use the most adequate and validated tool (de Vet et al., 2011).

Malnutrition is also known as a risk factor for falls (Eglseer et al., 2020; Meijers et al., 2012). Although falls were the second most frequently studied nursing‐sensitive indicator identified in this review (Brett et al., 2020; Burton et al., 2018; Doran et al., 2013; Meijers et al., 2012; Meyer et al., 2020; Neziraj et al., 2021; Rocha do Carmo et al., 2020; Schildmeijer et al., 2018; Vikman et al., 2011), only the study by Neziraj et al. (2021) assessed the risk of falls in HC clients. The Downtown Fall Risk Index (DFRI) used to assess the risk of falls (Neziraj et al., 2021) is a valid instrument which was, however, developed for older people in hospital care and also tested for residential care (Rosendahl et al., 2003). The high prevalence of risk of falls (63.7% of the participants) emphasises the importance of assessing the risk of falls on a regular basis in order to initiate preventive interventions early on. Mobility impairment is reported to be the biggest risk factor for falls (Meijers et al., 2012) and, therefore, should be considered when assessing the risk of falls in HC clients. In addition, people often live in houses with steps, inadequate light or uneven flooring, which are factors associated with falls (Rocha do Carmo et al., 2020).

When comparing prevalence and incidence rates in home care between countries, one always has to consider, though, that HC differs across countries. Financing and regulatory mechanisms lead to different types and provision levels of services across countries, which results in different care systems (Genet et al., 2012). HC in Australia is provided and partly financed by the government and is structured in four packages, according to the needs of the clients (Department of Health, 2018). According to Xu et al. (2018), HC in China provides only assistance to clients and does not include case management or professional nursing services. In Sweden, the Social Service Act ensures that the inhabitants receive the assistance that is appropriate for their needs. The government and private institutions provide HC, and the size of the fee depends on the client's income. The assistance is individually assessed and customised (Peterson, 2017; Swedish Institute, 2021). A study by Carpenter et al. (2004) compared differences in HC across 11 European countries. HC varied widely with regard to the extent of formal care provision for HC receivers (lowest amount in Italy and highest amount in the United Kingdom) and impairment in activities of daily living and cognitive function (higher in France and Italy and lower in northern European countries; Carpenter et al., 2004). Differences in organisation and use of HC services as well as different amounts of time nurses spend with their clients and the method of data collection may influence variation in the prevalence rates.

### 4.1 Limitations

This literature review had some limitations. Although our literature search was conducted as systematically as possible, this review was not conducted as a systematic review, which might have led to more comprehensive results. We conducted a narrative review to obtain a rapid overview of the state of the art regarding the nursing‐sensitive indicators included in this review. The search for the literature was only carried out in two databases, which may have limited the number of identified studies. Another limitation is that we did not include only predefined measurement tools, whereby the data would have been more comparable. Due to the use of various measurement tools and different instruments, it is difficult to directly compare the rates. Furthermore, the validity and reliability of the included measurement tools were not taken into account. Nevertheless, this review is one of the first to give a comprehensive insight into several important and common nursing‐sensitive indicators.

### 4.2 Recommended for the future

Several measurement programmes and databases are available, such as the National Database of Nursing Quality Indicators (NDNQI®) (Health Links, 2015), the International Pressure Ulcer Prevalence™ survey (VanGilder et al., 2017) and the National Prevalence Measurement of Care Quality (LPZ study) (Halfens et al., 2013), but those studies do not focus on the HC setting. Therefore, it is recommended to apply standardised measurements to the HC setting because we also need standardised data collection in these settings. Especially, the prevalence of pain and incontinence among clients in HC should be examined more closely because data on these indicators are rare. It is vital to recognise that many HC clients have more than one care problem. In the study of Neziraj et al. (2021), 30% of the participants had two health risks, such as a risk of pressure injuries, a risk of malnutrition, or a risk of falls. As a result, nurses in HC should assess all risks for the common nursing‐sensitive indicators and initiate adequate evidence‐based interventions to prevent the occurrence of these nursing‐sensitive indicators. This may also enable people to stay at home for as long as possible, as we know, for example, that incontinence is a risk factor for placement in residential care (Northwood et al., 2021).

## 5 CONCLUSION

The results of this literature review show that the prevalence or incidence rates for falls, incontinence, malnutrition, pain, pressure injury and restraints in HC vary widely. Malnutrition and pain were identified as topics that were the most frequently investigated nursing‐sensitive outcomes. Due to the use of various measurement tools and different instruments, it is difficult to directly compare the rates. These findings lead us to recommend implementing standardised data collection in the HC setting to obtain an insight into prevalence rates, increase transparency and increase the quality of care provided.

## AUTHOR CONTRIBUTIONS

L.M. Lampersberger, S. Bauer, S. Osmancevic: Conception and design of the manuscript. L.M. Lampersberger: Data analysis. L.M. Lampersberger, S. Bauer, S. Osmancevic: Interpretation of data. L.M. Lampersberger, S. Osmancevic: Drafting the article. L.M. Lampersberger, S. Bauer, S. Osmancevic: Final approval of the version to be submitted.

## CONFLICT OF INTEREST

There was no conflict of interest in this study.

## Data Availability

Data sharing is not applicable to this article as no new data were generated or analyzed in this study.
